# Inorganic Compounds as Remineralizing Fillers in Dental Restorative Materials: Narrative Review

**DOI:** 10.3390/ijms24098295

**Published:** 2023-05-05

**Authors:** Leena Ibraheem Bin-Jardan, Dalal Ibrahim Almadani, Leen Saleh Almutairi, Hadi A. Almoabid, Mohammed A. Alessa, Khalid S. Almulhim, Rasha N. AlSheikh, Yousif A. Al-Dulaijan, Maria S. Ibrahim, Afnan O. Al-Zain, Abdulrahman A. Balhaddad

**Affiliations:** 1 College of Dentistry, Imam Abdulrahman Bin Faisal University, P.O. Box 1982, Dammam 31441, Saudi Arabia; leena1421@hotmail.com (L.I.B.-J.); dalalmad@outlook.com (D.I.A.); leenalmutairiiau@gmail.com (L.S.A.); hadi44aziz@gmail.com (H.A.A.); x.mae@hotmail.com (M.A.A.); 2 Department of Restorative Dental Sciences, College of Dentistry, Imam Abdulrahman Bin Faisal University, P.O. Box 1982, Dammam 31441, Saudi Arabia; ksalmulhim@iau.edu.sa (K.S.A.); ralsheikh@iau.edu.sa (R.N.A.); 3 Department of Substitute Dental Sciences, College of Dentistry, Imam Abdulrahman Bin Faisal University, P.O. Box 1982, Dammam 31441, Saudi Arabia; yaaldulaijan@iau.edu.sa; 4 Department of Preventive Dental Sciences, College of Dentistry, Imam Abdulrahman Bin Faisal University, P.O. Box 1982, Dammam 31441, Saudi Arabia; msibrahim@iau.edu.sa; 5 Restorative Dentistry Department, Faculty of Dentistry, King Abdulaziz University Jeddah, P.O. Box 80209, Jeddah 21589, Saudi Arabia; alzain@kau.edu.sa

**Keywords:** biofilm, bioactive, dental, secondary caries, resin composite

## Abstract

Secondary caries is one of the leading causes of resin-based dental restoration failure. It is initiated at the interface of an existing restoration and the restored tooth surface. It is mainly caused by an imbalance between two processes of mineral loss (demineralization) and mineral gain (remineralization). A plethora of evidence has explored incorporating several bioactive compounds into resin-based materials to prevent bacterial biofilm attachment and the onset of the disease. In this review, the most recent advances in the design of remineralizing compounds and their functionalization to different resin-based materials’ formulations were overviewed. Inorganic compounds, such as nano-sized amorphous calcium phosphate (NACP), calcium fluoride (CaF_2_), bioactive glass (BAG), hydroxyapatite (HA), fluorapatite (FA), and boron nitride (BN), displayed promising results concerning remineralization, and direct and indirect impact on biofilm growth. The effects of these compounds varied based on these compounds’ structure, the incorporated amount or percentage, and the intended clinical application. The remineralizing effects were presented as direct effects, such as an increase in the mineral content of the dental tissue, or indirect effects, such as an increase in the pH around the material. In some of the reported investigations, inorganic remineralizing compounds were combined with other bioactive agents, such as quaternary ammonium compounds (QACs), to maximize the remineralization outcomes and the antibacterial action against the cariogenic biofilms. The reviewed literature was mainly based on laboratory studies, highlighting the need to shift more toward testing the performance of these remineralizing compounds in clinical settings.

## 1. Introduction

Tooth decay, often known as dental caries, is a societal and pervasive disease that affects people of every age and every group of the population worldwide. It results from mineral loss from susceptible tooth structures due to the acidic challenge induced by the cariogenic microorganisms that are capable of fermenting dietary carbohydrates [1,2]. The current concepts also identify it as an infectious, non-contagious, dental biofilm-mediated, and dental plaque-dependent disease [1,2]. Dental biofilms contain acid-producing bacteria that interact with the residual food or fermentable carbohydrates to demineralize the tooth structure, causing dysbiosis in minerals comprising the tooth structure [3]. The affected imbalance of minerals, namely phosphate and calcium, causes mineral loss in teeth. Thus, the continuous mineral loss may create cavitations associated with a higher rate of clinical intervention, starting from simple fillings and the probability of turning into an endodontic treatment due to neglecting this disease at its initial stages [4,5].

Dental caries could be treated by different means to restore the lost tooth structure, using non-invasive operative approaches to arrest active non-cavitated carious lesions and invasive approaches by removing and replacing the defective tooth structure with dental restorations [6]. Despite the increased use of resin-based composite restorations as the most used restorative material, several inherent physical properties could influence the integrity of the restoration margins, including polymerization shrinkage, modulus of elasticity, solubility, and water sorption [7]. Due to the resultant stresses caused by polymerization shrinkage, these polymeric materials are susceptible to marginal integrity failure, staining, microleakage, biofilm accumulation, and secondary caries [8]. As a result, dental restorations must be checked periodically to monitor and intervene in any failure at its early stage. Individuals with dental restorations placed within 6–36 months could be classified as medium to high caries-risk patients, and frequent dental visits every 6 to 12 months are needed [9,10,11]. During these visits, patients are examined for the onset of new carious lesions. Furthermore, old restorations must be monitored for biological and mechanical failures. Mechanical and physical failure reveals less concern, as usually the restoration can be repaired conservatively [9,10,11]. Opposingly, biological failure, such as secondary caries at the tooth-restoration interface, presents more challenges, as removing the secondary lesions is usually associated with more tooth destruction [9,10,11].

## 2. Statement of the Problem

In restorative dentistry, secondary caries is a complication following the placement of restorations leading to its failure, especially in polymeric restorative materials [12]. It is a developing lesion at the margin of an existing restoration, a common area for plaque accumulation and biofilm development [12]. The process of developing secondary caries has the same notion of primary carious lesions on any sound tooth, starting with demineralization and imbalance of net minerals, and followed by enzymatic breakdown of the tooth structures. However, a restoration or sealant margin might modify this process and assist in further tooth destruction [13]. In previous studies, the prevalence of secondary caries after polymeric restorative materials placement was as high as 60%. It was identified as the leading cause of resin-based composite degradation and replacement [14]. Secondary caries’ existence around the margins is characteristically represented in two regions: the surface lesion; which grows perpendicularly to the tooth’s surface adjacent to a restoration; and wall lesion, which extends perpendicularly along the interface of tooth/restoration [15].

It is reported that the caries-related bacteria in human saliva that induce both primary and secondary caries are comparable, most frequently *Streptococcus mutans*, *Lactobacilli*, and *Actinomyces naeslundii* [16]. The complexity and cariogenicity of oral biofilms contribute to the difficulty in developing effective restorative materials to render secondary caries [17,18]. Nonetheless, changes surrounding the resin-based composite restoration allow for the passage of salivary proteins and liquids saturated with foreign particles, rendering restorations to degrade biologically [19]. Esterase in human saliva can aggressively degrade ester groups in resin-based composites, accumulating monomer byproducts that may encourage biofilm growth [20]. When materials deteriorate and release their integrated agents, there are tremendous impacts on their long-term mechanical and physical performance [14]. Furthermore, surface features, such as surface roughness, significantly affect the adhesion phase of dental biofilms [14,21].

Throughout the restoration lifespan, under certain factors at the tooth-restoration interface, cycles of two contrary mineral loss and gain processes occur in demineralization and remineralization [22,23,24,25,26]. Understanding the mechanism of these two processes is essential when developing advanced approaches to control the onset of primary and secondary caries. The main participants in these cycles are calcium (Ca^2+^), phosphate (PO_4_^3−^), and fluoride (F^−^) ions. The imbalance between pathological and protective factors may lead to more mineral loss and progression of dental caries [22,23,24,25,26], considering that the tooth-restoration interface is a plaque-stagnation area. Demineralization is the process of mineral loss from the tooth surface that starts and usually grows in the presence of intrinsic and extrinsic modifiable risk factors [26]. Acidic attack is the leading cause of the chemical demineralization of teeth, which happens in two ways—dietary acids and microbial attack [26,27,28,29].

Similar to primary carious lesions, demineralization begins when the bacteria metabolize fermentable carbohydrates, producing organic acids that spread around nano-sized aqueous spaces between the hexagonal crystal of the tooth [25,30,31]. This process is followed by dissolving the Ca^2+^ and PO_4_^3−^ ions into those spaces, which leads to a much more acid-soluble structure than pure hydroxyapatite, resulting in the substitution of phosphate ions for carbonate ions in the crystal lattice [25,30,31]. Such substitution can produce defects and calcium-deficient regions, which present clinically as white spots [25,32,33,34]. One of the most critical factors in that dynamic process is the oral pH. The pH required to initiate mineral loss in root dentin ranges between 6–6.8, compared to 5.4 in the enamel [3,35], indicating that dentin and cementum are more acid soluble than enamel due to their higher magnesium and carbonate contents [3,35].

## 3. Remineralizing Dental Materials as a Strategy to Prevent Secondary Caries

The remineralization or the repairing process can be achieved to overcome the challenge of demineralization, either by host-related factors or clinical intervention. Remineralization has been studied for many decades, in an attempt to understand the mechanism and develop technologies that could help reverse incipient caries and prevent tooth demineralization [36]. Fluoride (F) has long been known to be effective in preventing caries by reducing tooth dissolution and enhancing tooth remineralization processes [36]. Fluoride has been seen in studies to directly deposit fluorapatite (FA) or fluoridated hydroxyapatite (FHA) over the affected tooth surface or to promote the transformation of other calcium phosphate phases to FA or FHA. As a result, FA and FHA formation can reduce the solubility of enamel and dentin [36,37,38]. However, studies had shown that the most significant effect was obtained only when 50% of the hydroxyl groups were replaced with fluoride, corresponding to the greatest lattice stability and low lattice-free energy [36,37,38].

Ca^2+^ and PO_4_^3−^ ions, bioactive glass (BAG), and boron nitride (BN) can also be utilized in the remineralization process by depositing a hydroxyapatite layer over the affected tooth surface [36,39,40]. In comparing Ca with F ions, in vitro, mechanistic studies have shown that calcium is approximately twenty times more potent than phosphate in inhibiting enamel dissolution [36,39,40]. Nowadays, nanotechnology is one of the most inventive concepts, which has shown significant success. Nanomaterials exhibit superior antimicrobial activity and comparable physical properties compared to conventional materials [41,42,43,44,45]. This is most likely due to the nanoparticles’ small size and high surface area, which can release high levels of ions at a low filler level [44,45,46]. Therefore, remineralizing nano-fillers in dental restorative materials can be an effective strategy to prevent secondary caries at the tooth-restoration interface. The release of ions from the restorations can prevent oral biofilm attachment and favor the process of remineralization at a specific micro-level site [41,45].

Until now, most restorative polymeric materials have had no bioactivity, exaggerating the risk of secondary caries development around resin-based composite restorations, which are considered a major limitation to the current treatment approach. To address these concerns, researchers have concentrated their efforts on designing antibacterial features that can reduce bacterial attachment to prevent further accumulation of biofilms and hinder demineralization [2,14,47]. The potential of a dental material to influence its biological environment favorably offers a way to extend longevity and clinical performance inside the oral cavity [47]. The advantages of integrating bioactive chemicals in dental polymeric formulations, which are thought to be essential for effectively managing caries around restorations, are currently a hot-spot area in dentistry. This review discusses the applications and functionalization of different remineralization approaches and their uses in different restorative materials. This narrative review focused on including only remineralizing compounds in different restorative materials. Other bioactive compounds, such as quaternary ammonium, organic agents, metallic particles, and nanotubes, with antibacterial properties, were not included as they were reviewed in some of the authors’ previous papers [14,48]. All articles in English with no specific time frame were extracted from PubMed and Scopus and included in the review.

## 4. Remineralizing Fillers in Restorative Dental Materials

Several studies investigated incorporating different bioactive and antibacterial compounds into the resin matrix system to limit the onset of secondary caries. Two main approaches have been heavily explored; (1) the incorporation of antibacterial compounds or particles, and (2) the use of a remineralization approach that can neutralize the acidity induced by the oral biofilms and interfere with biofilm growth [47].

**Figure 1:**
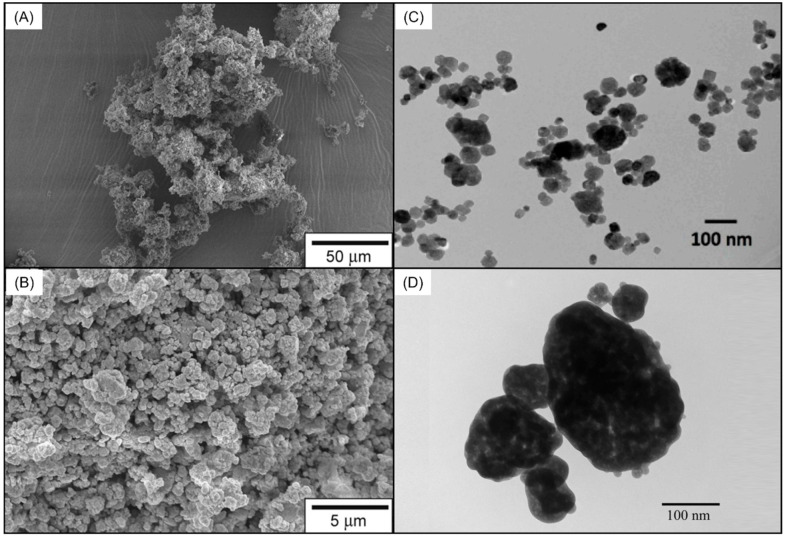
Different compounds that were incorporated in different resin-based materials to impart bioactivity and remineralize the surrounding dental tissues subjected to demineralization. (**A**,**B**) Scanning electron micrograph showing bioactive glass particles. Reprinted/adapted with permission from Ref. [49]. 2016, © Elsevier. Transmission electron microscope illustrating the size of (**C**) calcium fluoride (CaF_2_) nanoparticles. Reprinted/adapted with permission from Ref. [50]. 2020, Mitwalli et al. Another transmission electron microscope illustrating the size of (**D**) nano-sized amorphous calcium phosphate (NACP) fillers. Reprinted/adapted with permission from Ref. [51]. 2022, © Elsevier.

In the second approach, which is the focus of this review, several compounds have been implemented in different restorative materials (Figure 1), which are Nano-sized Amorphous Calcium Phosphate (NACP), Calcium Fluoride (CaF_2_), Bioactive Glass (BAG), Hydroxyapatite (HA), Fluorapatite (FA), and Boron Nitride (BN). While the primary effect of these compounds relies on their ability to neutralize the acidity induced by the attached cariogenic biofilms and favor the remineralization process, their indirect biofilm inhibition has been observed in several studies, showing dual benefits in modulating the oral biofilms.

### 4.1. Nano-Sized Amorphous Calcium Phosphate (NACP) Fillers

The use of materials that release Ca^2+^ and PO_4_^3−^ ions is suggested to support the remineralization process. It is well known that Ca^2+^ and PO_4_^3−^ ions have multiple applications in dentistry since these two components form the inorganic portion of human teeth and bones [2]. The bioactivity of calcium phosphate (CaP) phases in dental materials has been examined. Different CaP compounds have been considered with a variation on the Ca/P molar ratio and the salt phase stability, including dicalcium phosphate dihydrate (DCPD), dicalcium phosphate anhydrous (DCPA), tetracalcium phosphate (TTCP), tricalcium phosphate (TCP) and amorphous calcium phosphate (ACP) [2,47,52,53,54]. Amorphous calcium phosphate (ACP) is the first phase that formed before reaching hydroxyapatite (HA), which is known to be the final thermodynamically stable product. ACP’s lack of crystallinity structure, and it has high solubility, indicating that it contains a distinguished structure among CaP [2]. Nowadays, NACPs have been introduced with about a 100 nm particle size. These particles can neutralize the acidic environment caused by cariogenic bacteria and adjust the oral pH, subsequently promoting the remineralization process [55,56].

Historically, several resin-based materials containing calcium orthophosphate phases (DCPA, DCPD, ACP, and TCP) were investigated [57]. In enamel lesions, results showed that a resin-based composite with ACP-releasing ions at 40 wt.% released 0.74 mmol\L of calcium and 0.54 mmol/L of phosphate ions [57]. These released ions participated in remineralizing 14% of enamel lesions in a period of 30 days in comparison with another material containing commercial fluoride cement, which achieved 4% of remineralization in the same period [57]. Another study showed that in a period of 5 weeks, resin cement comprising DCPA and TTCP at 73–78 wt.% released 0.05–0.1 mmol/L of phosphate as well as 0.3–0.5 mmol/L of calcium, which were capable of remineralizing the demineralized dentin lesions by 38–47% [57]. The main drawback observed in these compounds was the low amount of ion release and the inferior mechanical properties [2]. Thus, nanotechnology via NACP was advanced enough to overcome these drawbacks and tailor different restorative materials with a high amount of ion release and excellent mechanical properties.

#### 4.1.1. NACP in Resin-Based Composite Restorations

NACP synthesized via the spray-drying technique was heavily evaluated in multiple studies. Early investigations compared different weight percentages of NACP in resin-based composite formulations [56,58]. Resin-based composite, composed of bisphenol glycidyl dimethacrylate (BisGMA), triethylene glycol dimethacrylate (TEGDMA), and glass fillers, was modified to contain 10 to 40 wt.% of NACP [56]. The flexural strength value was reduced as the NACP concentration increased. Still, the values of the formulations were higher than 80 MPa, the minimum suggested by the International Organization for Standardization (ISO). It was found that the 10 wt.% NACP resin-based composite demonstrated reduced Ca^2+^ and PO_4_^3−^ ion release compared to the other formulations [56]. The same was observed in another study, where 10 and 15 wt.% NACP resin-based composite did not reveal a high ion release [58]. As a result, recent investigations designing NACP resin-based composites have focused on incorporating 20 wt.% or more of NACP into resin-based composite formulations.

In one study, using BisGMA and TEGDMA as a resin matrix system, 30 wt.% of NACP and 35 wt.% of glass fillers were mixed with and without different small fractions of silver nanoparticles [59]. It was found that combining NACP and silver nanoparticles resulted in a significant inhibition against multi-species biofilm formation, bacterial metabolic activities, and lactic acid production [59]. An in situ experiment was held in 2013 to test the NACP resin-based composite inside the oral cavity [60]. Resin-based composites containing 20 wt.% of NACP were used to fill cavities prepared in extracted bovine teeth and mounted in removable appliances. Participants placed the appliances inside their oral cavities for 14 weeks. Then, the biofilm formation and mineral loss of the enamel surface at the tooth restoration interface via transverse microradiography were assessed. Less biofilm formation, but not statistically significant, was observed over the NACP resin-based composite with a higher amount of Ca^2+^ and PO_4_^3−^ ions in the biofilms. Regarding the mineral loss, the lesion depth around the NACP resin-based composite was significantly less than the control group [60]. Such findings may reveal the capabilities of NACP resin-based composites to hinder enamel demineralization at the tooth-restoration interface.

The ability of NACP resin-based composites to recharge the resin matrix with Ca^2+^ and PO_4_^3−^ ions to allow frequent ion release is a primary concern. While it is worth saying that the ion release process will be initiated only with low pH at the risk of demineralization, having rechargeable NACP resin-based composites will be beneficial to assure long-term bioactivity [47], especially among high caries risk patients. The rechargeable resin matrix was composed of pyromellitic glycerol dimethacrylate (PMGDM) and ethoxylated bisphenol A dimethacrylate (EBPADMA) at a 1:1 ratio [61,62]. The amount of Ca^2+^ and PO_4_^3−^ ion re-release was significantly higher among different cycles of recharges compared to the NACP resin-based composite that contained BisGMA and TEGDMA as a resin matrix [61]. When the NACP rechargeable resin-based composite was combined with DMAHDM, a significant biofilm reduction of 2- to 3-log was observed [62]. The synergetic antibacterial and remineralization action was also observed when NACP was mixed with a protein-repelling agent, named methacryloyloxyethyl phosphorylcholine (MPC) [52]. However, some of the main drawbacks of this rechargeable resin matrix are the reduced mechanical properties compared to other formulations [61,62], suggesting the need for further characterization to enforce the strength of this formulation.

In most of the earliest investigations, the synergistic effect of 20% NACP and DMAHDM was not reflected. However, increasing the DMAHDM concentration from 3 to 5 wt.% allowed the synergistic effect of these two bioactive compounds to be clearly observed. Increasing the DMAHDM concentration was associated with an increased surface charge density by around 2-fold [63], allowing the resin-based composite to interact more aggressively with the bacterial membrane. Adding the NACP to a resin-based composite containing 3 and 5% of DMAHDM resulted in a 1-log additional reduction compared to the formulations with no NACP. The overall biofilm decrease in total microorganisms, total *streptococci*, total *lactobacilli*, and *mutans streptococci* compared to the control was a reduction of around 2- to 5-log. Significant inhibition was also observed concerning these formulations’ metabolic activities and lactic acid production [63]. This strong synergetic effect of 20% NACP and 3–5% DMAHDM was also potent after one year of water aging. The mechanical and antibacterial properties were slightly reduced but still sustained after aging [64].

**Figure 2:**
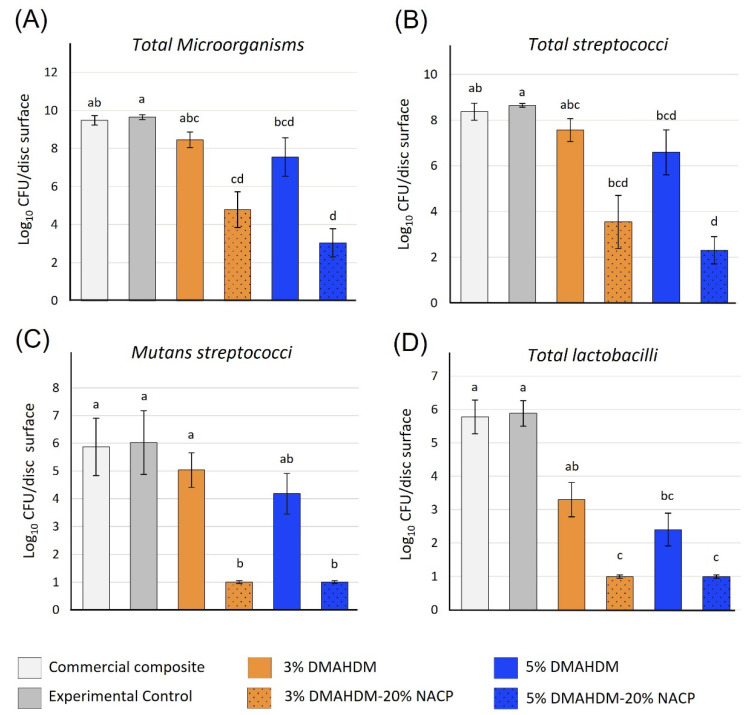
The biofilm inhibition of the NACP-DMAHDM against (**A**) Total microorganisms, (**B**) Total streptococci, (**C**) mutans streptococci, and (**D**) Total lactobacilli. More biofilm inhibition of 4- to 6-log reduction was observed when the DMAHDM was combined with the nano-sized amorphous calcium phosphate (NACP) fillers. Values indicated by different letters are statistically different from each other (*p* < 0.05). Reprinted/adapted with permission from Ref. [65]. 2020, Balhaddad et al.

**Figure 3:**
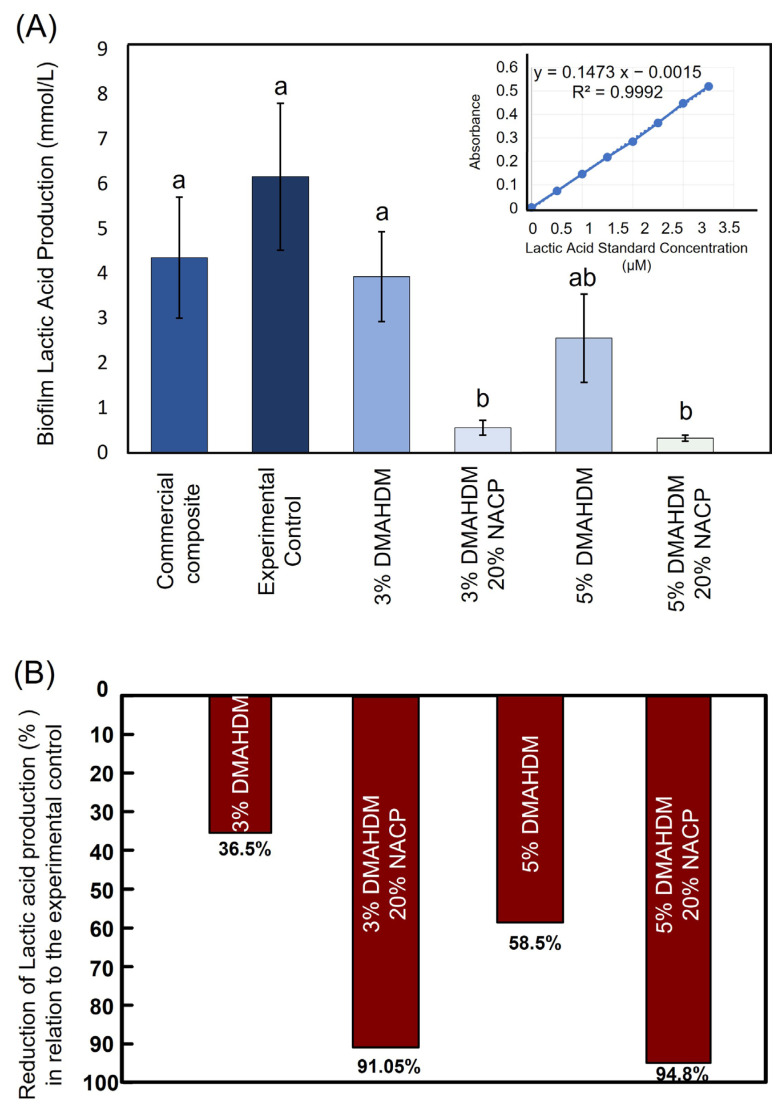
(**A**) The amount of lactic acid produced by multi-species cariogenic biofilms. Higher values indicate more lactic acid production. Values indicated by different letters are statistically different from each other (*p* < 0.05). (**B**) The percentage of lactic acid production inhibition shows the capabilities of nano-sized amorphous calcium phosphate (NACP) fillers to prevent demineralization and promote the remineralization process. Reprinted/adapted with permission from ref. [65]. 2020, Balhaddad et al.

This formulation with an increased DMAHDM concentration was investigated against highly cariogenic multi-species plaque-derived biofilms transferred from root carious lesions [65]. Resin-based composites containing 3 and 5% of DMAHDM without NACP were able to inhibit total microorganisms, total *streptococci*, total *lactobacilli*, and *mutans streptococci* by only 2-log compared to the control. When the NACP was added (Figure 2), significant inhibition of 4- to 6-log reduction was achieved, emphasizing the potency of this combination. The same trend was observed when the lactic acid production was quantified [65]. Incorporating DMAHDM alone inhibited lactic acid production by 36 to 58%. When NACP was combined with DMAHDM, a reduction in lactic acid production of more than a 90% was observed (Figure 3). In this study, it was found that the incorporation of neither DMAHDM nor NACP influenced the degree of conversion of the synthesized formulations [65].

Similar findings were observed when the NACP-DMAHDM resin-based composites were challenged using anaerobic biofilms isolated from deep periodontal pockets [66]. In this study, the DMAHDM-NACP resin-based composites substantially restrained the growth of anaerobic microorganisms by 3- to 5-log. Additionally, significant inhibition was seen when the metabolic activities and polysaccharide production were measured [66]. Such findings may suggest that these formulations can prevent the growth of periodontal pathogens around the margins of subgingival extending restorations.

Recent investigations evaluated the incorporation of NACP into a low-shrinkage-stress resin matrix consisting of urethane dimethacrylate (UDMA) and triethylene glycol divinylbenzyl ether (TEG-DVBE) [67,68]. This formulation was designed to minimize the stress induced by the resin shrinkage at the tooth-restoration interface. This formulation effectively inhibited the *S. mutans* biofilms without affecting the restoration’s mechanical properties and the polymerization kinetics [67]. The high amount of Ca^2+^ and PO_4_^3−^ ion release could significantly preserve the enamel microhardness after microbial demineralization compared to the control samples with no NACP [67]. This approach with a high concentration of DMAHDM up to 5% was capable of inducing potent antibacterial action against multi-species biofilm, resulting in a 2- to 5-log biofilm reduction [68]. The antibacterial activity was sustained after 20,000 cycles of thermocycling aging, equivalent to two years of clinical service [68]. These findings suggest that this combinatory approach of DMAHDM and NACP can preserve its bioactivity after aging.

#### 4.1.2. NACP in Resin-Based Pit and Fissure Sealants

Dental sealants serve as a protective physical barrier against plaque accumulation in deep pits and fissures in the occlusal surface of teeth [17]. Pit and fissure sealants can therefore successfully prevent cavities and reduce the need for future restorations. The therapeutic bioactivity of resin-based pit and fissure sealants could be greatly enhanced and lead to caries prevention by using remineralizing agents [17]. Ibrahim et al. conducted a series of investigations to evaluate the mechanical and bioactive properties of dental sealants containing different mass fractions of NACP and 5 wt.% DMAHDM. It was found that sealants containing 20 wt.% of NACP and 5 wt.% of DMAHDM demonstrated massive Ca^2+^ and PO_4_^3−^ ion release without compromising the mechanical properties of the sealant [69]. The unique resin matrix compositions, containing PMGDM, EBPADMA, BisGMA, and 2-hydroxyethyl methacrylate (HEMA), permitted frequent cycles of ion recharge and release, allowing long-term bioactivity of the formulation [69].

**Figure 4:**
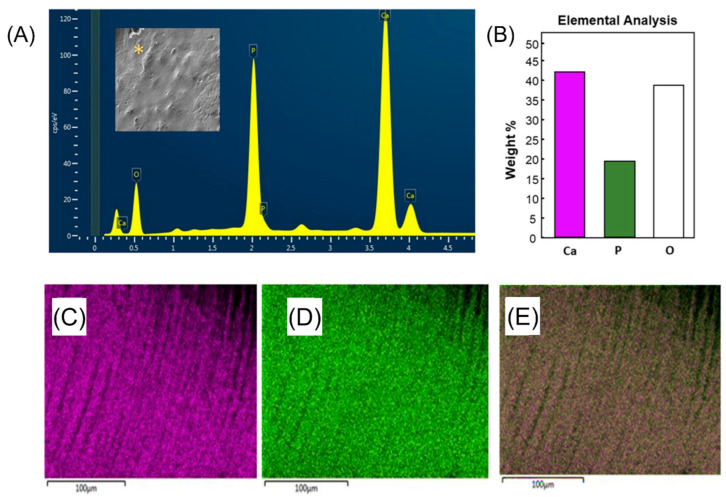
Scanning electron microscopy/energy-dispersive X-ray spectroscopy (SEM-EDX) illustrating the mineral contents of enamel restored with pit and fissure sealant containing nano-sized amorphous calcium phosphate (NACP) fillers. (**A**) SEM-EDX spectrum highlighting the mineral contents of the enamel surface restored with NACP-containing sealant. (**B**) Percentage of elemental concentration in weight of calcium, phosphate, and oxygen within the enamel restored with NACP-containing sealant. EDX mapping of elemental (**C**) calcium and (**D**) phosphate. (**E**) EDX mapping shows the overlay of C and D images, where the calcium is indicated in the pink color, and the phosphate is displayed in the green color. Reprinted/adapted with permission from Ref. [72], 2020, © Elsevier.

**Figure 5:**
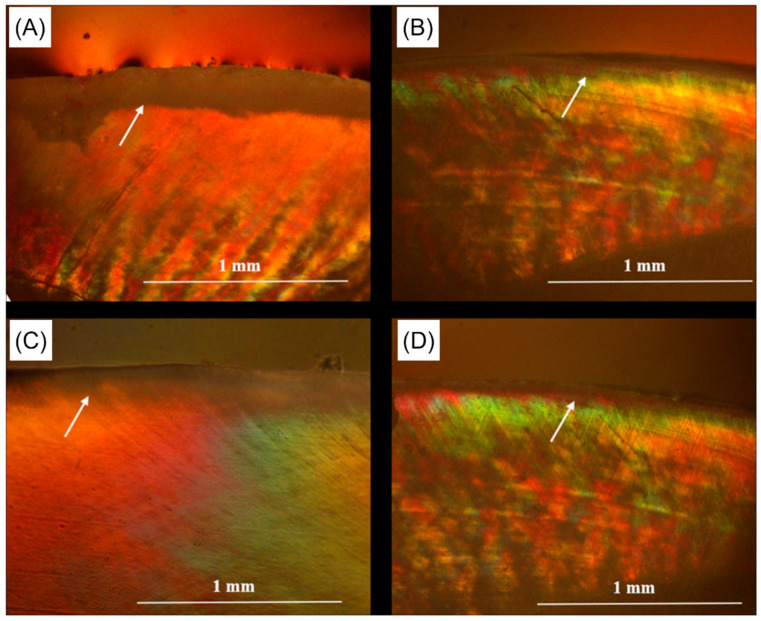
Polarized light photomicrograph showing dark bands (white arrows) at the enamel surface restored with parental resin-based sealant (**A**,**B**) and NAC-containing resin-based sealants (**C**,**D**), which represent the demineralized areas. It can be observed that enamel restored with the NAC-containing resin-based sealants is associated with a narrow dark band, compared to the wide one in the control group, suggesting the capabilities of these bioactive resin-based sealants to resist demineralization and mineral loss. Reprinted/adapted with permission from Ref. [72], 2020, © Elsevier.

The exact formulation of 20 wt.% NACP and 5% DMAHDM was challenged with *S. mutans* biofilms [70]. Only when combined with DMAHDM did NACP-containing sealants reduced the *S. mutans* biofilm by around 4-log. The cariogenic biofilm’s metabolic activities, lactic acid, and polysaccharide production were also significantly reduced [70]. The same formulation was also found effective in eradicating *Candida albicans* growth and activities [71], one of the possible contributing microorganisms in the pathogenesis of early childhood caries. Qualitative analyses via scanning electron microscopy/energy-dispersive X-ray spectroscopy (SEM-EDX) revealed that enamel surfaces restored with NACP-containing sealant revealed higher microhardness and presented more elevated Ca^2+^ and PO_4_^3−^ ions following chemical demineralization (Figure 4). Furthermore, polarized light microscopy (PLM) images showed less demineralized surface area around the enamel restored with NACP-containing sealant than the control (Figure 5) [72]. In a more challenging condition, saliva-derived biofilms secluded from high-caries risk pediatric patients were grown over the sealants [73]. Using sealants containing NACP and DMAHDM, biofilm development, lactic acid production, and metabolic activities were all significantly reduced [73].

#### 4.1.3. NACP in Dental Adhesives

Resin-based adhesives with remineralizing properties decrease the risk of white spots and secondary caries around orthodontic brackets and dental restorations. NACP as adhesive fillers is a promising approach to limit such consequences by releasing significant amounts of Ca^2+^ and PO_4_^3−^ ions [74]. A study found that a 30% NACP resin-based composite prevented an acid attack and increased the pH of the solution from a pH of 4 which is cariogenic, to a safe pH of 6.5, most likely due to NACP alkaline nature [74]. The bonding agent is predicted to benefit from the 30% NACP inclusion in the form of antibacterial, acid neutralization, and remineralizing properties [74]. When conjugated with silver nanoparticles, NACP-containing adhesive reduced the biofilm growth, metabolic activities, and lactic acid production of multi-species cariogenic biofilms by more than 50% [74]. In another investigation, NACP-containing adhesives with and without DMAHDM preserved the bonding strength after 12 months of aging, greater than what was observed in the control groups [75]. The DMAHDM was inserted in the dental adhesive to convey antibacterial properties and the remineralization capabilities induced by the NACP fillers [75].

Using a rechargeable resin matrix in dental adhesives was also attempted [76,77]. When combined with MPC or DMAHDM, rechargeable NACP dental adhesives were found to release high amounts of Ca^2+^ and PO_4_^3−^ ions in low pH and inhibit the growth and activities of highly cariogenic biofilms [76,77]. It is worth saying that reachability is less critical among dental adhesives than resin-based restorations or sealants, as the adhesive layer is usually sealed and protected from the external environment. In a recent interesting investigation, the bonding strength of an NACP-containing adhesive was significantly improved by imparting magnetic particles and using a magnetic field during the bonding procedure [78]. The bonding strength was increased by around 30–40% compared to the control. When DMAHDM as an antibacterial monomer was added, the designed adhesive demonstrated high bonding strength with excellent antibacterial and remineralization properties [78]. These findings may encourage using a combinatory approach to creating highly bioactive resin-based materials with several desirable properties.

Moreover, a study compared the effect of an NACP-containing adhesive with a control group in a challenging condition with an *S. mutans* biofilm [79]. Results showed that the control group achieved minimal remineralization, while the NACP-containing adhesive achieved a high rate of remineralization via the massive release of Ca^2+^ and PO_4_^3−^ ions. Furthermore, the NACP-containing adhesive reduced the lactic acid production and minimized the biofilm growth of the *S. mutans* biofilm [79]. The performance of the NACP dental adhesive was also investigated using dentin as a bonding substrate [80].

The growth of white spot lesions surrounding orthodontic brackets is one of the biggest obstacles during orthodontic treatment [81]. The onset of these lesions compromises the mechanical properties and the esthetic appearance of teeth [81]. The incorporation of 40 wt.% of NACP into an orthodontic adhesive was attempted [82]. When this adhesive was applied to attach orthodontic brackets to premolars, the bonding strength was comparable to the control. Furthermore, when an antibacterial monomer, named 2-methacryloxylethyl dodecyl methyl ammonium bromide (MAE-DB), was added to the NACP-adhesive, the orthodontic adhesive reduced the growth of *S. mutans* biofilms and preserved the enamel microhardness [82]. Such an approach could be beneficial to minimize the onset of white spot lesions around orthodontic brackets.

#### 4.1.4. NACP in Resin-Based Dental Cements

Resin-based dental cements are used to bond or lute indirect or fixed restorations, such as crowns and bridges, to the tooth structure. Secondary caries around fixed restorations is a clinical concern, especially when these restorations are close to the gingival margins [83]. The onset of secondary caries affects the clinical longevity of the placed restorations, leading to the restorations’ replacement and sometimes tooth extraction [84]. Therefore, imparting bioactive restorative materials into dental cements may minimize the biological failure of fixed restorations. In one study, a resin-based dental cement was designed to contain 25 wt.% of NACP and different mass fractions of DMAHDM, ranging between 3–5 wt.% [85]. The bonding strength, flexural strength, elastic modulus, and film thickness of the designed formulations were comparable to the control. Additionally, a high amount of Ca^2+^ and PO_4_^3−^ ion release was observed. When the formulations were challenged with *S. mutans* biofilms, a reduction in 3-log was noted when the NACP was combined with 5% DMAHDM [85].

The rechargeability of bioactive resin-based cement was achieved in another investigation, where the resin matrix was composed of PMGDM and EBPADMA [86]. Several cycles of recharge and re-release were achieved with an excellent amount of Ca^2+^ and PO_4_^3−^ ion release. This rechargeable formulation also effectively inhibited the growth and activities of *S. mutans* biofilms [86]. These findings may embark on new avenues to minimize the failure of indirect restorations due to secondary caries.

### 4.2. Calcium Fluoride (CaF_2_) Fillers

Fluoride-releasing dental materials have been frequently studied and used in dentistry due to their working mechanisms, which significantly affect the progression of dental caries [87,88,89]. Caries prevention is achieved through the adsorption of fluoride ions, which occurs on HA crystals’ surfaces, which in turn, prevent crystals dissolution in an acidic cariogenic medium [87,90]. Fluoride ions fight dental caries through acid resistance, fluorapatite formation, inhibition of bacterial growth in the oral cavity, remineralization process promotion, and demineralization process inhibition [87,91,92,93]. The addition of fluoride to resin-based materials can promote the prevention of secondary caries formation; it presents in different addition forms, such as organic fluoride, inorganic salts, and leachable glasses. Sodium fluoride (NaF) and Tin(II) fluoride (SnF_2_) have been used as water-soluble salts, and more recently, CaF_2_ particles have been used [87,91,92,93,94]. Due to their functions as labile reservoirs for the calcium (Ca^2+^) and fluoride (F^−^) ions and their ability to enhance the remineralization effects of the F regimen without increasing the F level, CaF_2_ particles are of great interest in the prevention of dental caries.

#### 4.2.1. CaF_2_ in Resin-Based Composite Restorations

**Figure 6:**
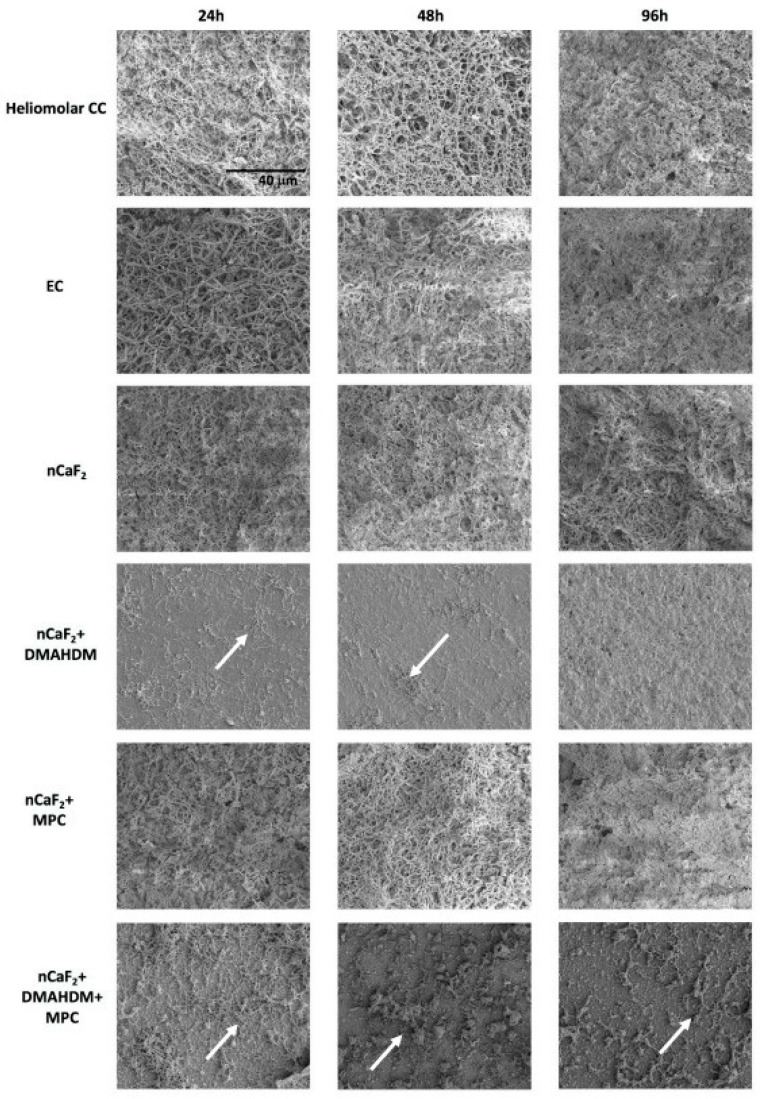
Scanning electron microscope images showing the growth of cariogenic multi-species saliva-derived biofilms over different bioactive resin-based composites. White arrows are showing the biofilm colonies over the resin-based composites. Combining calcium fluoride nanoparticles and DMAHDM was associated with the least biofilm formation. Reprinted/adapted with permission from Ref. [50]. 2020, Mitwalli et al.

Incorporating calcium fluoride (CaF_2_) particles into a resin-based composite was attempted in several investigations. CaF_2_ particles at the load of 30% were associated with a high amount of Ca^2+^ and F^−^ ion release without affecting the material’s mechanical properties [95]. In another investigation, 15 wt.% of CaF_2_ nanoparticles were incorporated into a resin-based composite system containing BisGMA and TEGDMA as a resin matrix and glass as co-fillers [50]. This formulation was achieved with and without the addition of DMAHDM and MPC. When CaF_2_ nanoparticles were incorporated into the parental formulation, good mechanical properties were achieved. However, adding either DMAHDM or MPC to the formulation significantly reduced the mechanical properties, but the values were comparable to the commercial control. All the designed formulations were associated with a high amount of F^−^ and Ca^2+^ ion release, showing the remineralization potential of these formulations. When the antibacterial properties were assessed, CaF_2_ nanoparticles were associated with minor antibacterial properties, but when the DMAHDM was added, significant antibacterial performance was observed (Figure 6). The bioactive formulation containing CaF_2_ nanoparticles and DMAHDM reduced the multi-species biofilm by around 4-log, illustrating this formulation’s potent antibacterial and remineralization capabilities [50].

One study functionalized CaF_2_ nanoparticles into a rechargeable resin-based composite matrix to allow the long-term release of ions [96]. The resin matrix was composed of PMGDM and EBPADMA. This formulation allowed multiple cycles of ion release and recharge, demonstrating that long-term ion release is possible. The only concerns related to this formulation are the reduced mechanical properties compared to the other resin matrix formulations, indicating the need for further characterization to engineer a mechanically sustained rechargeable formulation [96]. The synergetic effect of CaF_2_ nanoparticles and chlorhexidine was investigated in one study, where a 3-log inhibition against *S. mutans* biofilms was observed [97] and the metabolic activities and lactic acid production were considerably diminished [97]. Current findings suggest that CaF_2_ nanoparticles may contribute to remineralizing the tooth structure and reduce the amount of lactic acid production induced by the cariogenic species. The significant inhibition related to the biofilm colony-forming units (CFUs) or metabolic activities was only observed when the CaF_2_ nanoparticles were combined with another bioactive agent. Such findings suggest using several bioactive materials to maximize the protection of restored teeth.

#### 4.2.2. CaF_2_ in Resin-Based Pit and Fissure Sealants

For many years in preventive dentistry, dental practitioners have used glass ionomer as a fluoride-releasing sealant to occlude food stagnation areas and release F^−^ ions to the sealed tooth structure [98,99]. The main limitations of glass ionomer sealants were related to the low mechanical performance and the low retention rate. Therefore, highly flowable resin-based sealants were also used as an alternative material [98,99]. One of the main limitations of resin-based sealants is the lack of bioactivity and the development of carious lesions around the sealants. As a result, imparting bioactive compounds to resin-based sealants is a promising strategy to overcome this limitation.

One study attempted to incorporate CaF_2_ nanoparticles into a resin-based sealant at a load of 20 wt.% [100]. The resin matrix was composed of BisGMA and TEGDMA at the ratio of 1:1. The fillers were composed of barium boro-aluminosilicate glass particles, which were silanized with 4% 3-methacryloxypropyltrimethoxysilane, 2% n-propylamine, and CaF_2_ nanoparticles. DMAHDM was added to the formulation at 5 wt.%. The overall matrix-to-filler ratio was 1:1. Adding CaF_2_ nanoparticles and DMAHDM did not compromise the design formulations’ flowability and enamel shear bond strength. A significant F^−^ ion release was observed, and the amount of ion release was slightly reduced when DMAHDM was added to the formulation. Combining the CaF_2_ nanoparticles and DMAHDM significantly reduced the CFUs, metabolic activities, and lactic acid production of the *S. mutans* biofilms [100]. Such formulation may minimize the onset of caries around sealants and assure good mechanical and bonding properties during the clinical service inside the oral cavity.

#### 4.2.3. CaF_2_ in Dental Adhesives

A few investigations discussed the design of CaF_2_-dental adhesives. The addition of CaF_2_ fillers allowed dental adhesives to release a high amount of F^−^ ions without compromising the bonding strength of the material [101]. In another investigation, a dental adhesive containing zinc, calcium, fluoride, and bioglass compounds was engineered [102]. This bioactive adhesive demonstrated good bonding properties and polymerization kinetics with a high amount of ion release, revealing the high potential to remineralize tooth structure subjected to demineralization attacks. The zinc-calcium-fluoride-bioglass adhesive inhibited the *S. mutans* biofilms [102].

Incorporating CaF_2_ nanoparticles in an orthodontic adhesive as a remineralizing agent to prevent the onset of white spot lesions was attempted [103]. This incorporation was achieved in two different resin matrix systems. The first was composed of HEMA and BisGMA. In contrast, the other was composed of PMGDM and EBPADMA, both at the ratio of 1:1. The two dental adhesives released a high amount of F^−^ ions with a high amount of re-release upon recharge compared to a resin-modified glass ionomer (RMGI). All the formulations did not induce high cytotoxic effects against human gingival fibroblasts. The only concern is the reduced enamel bonding strength compared to the control, which may necessitate further characterization in future investigations [103].

#### 4.2.4. CaF_2_ in Resin-Based Dental Cements

**Figure 7:**
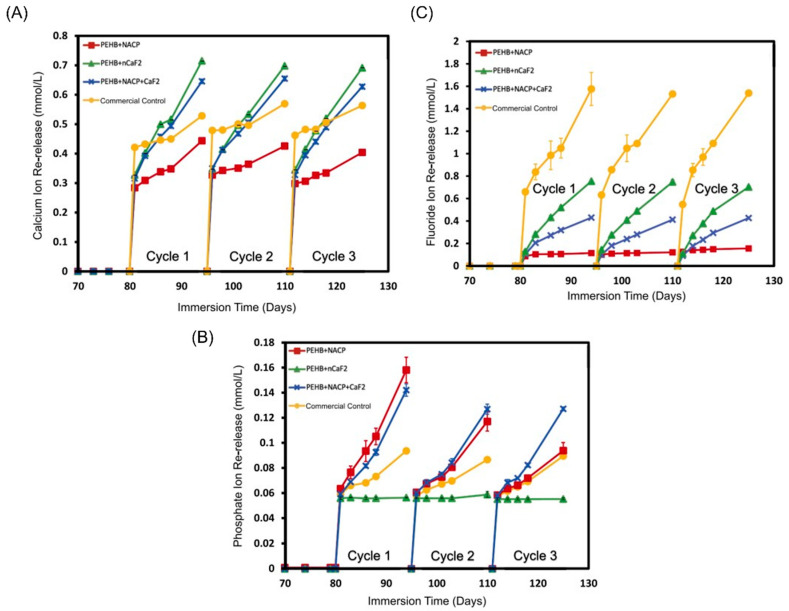
Resin-based dental cement containing 25 wt.% of calcium fluoride (CaF_2_) or 12.5 wt.% of CaF_2_ and 12.5 wt.% of nano-sized amorphous calcium phosphate (NACP) fillers, releasing high amounts of (**A**) calcium, (**B**) phosphate, and (**C**) fluoride ions. After 80 days of continuous release, the remineralizing resin-based cements were recharged for three consecutive cycles, indicating their capabilities of long-term clinical service inside the oral cavity. Reprinted/adapted with permission from Ref. [104]. 2022, © Elsevier.

The incorporation of CaF_2_ nanoparticles into fixed prostheses cement was achieved in one study [104]. The resin matrix was composed of PMGDM and EBPADMA, both at a ratio of 1:1 to allow rechargeability features. CaF_2_ nanoparticles were incorporated either alone at the load of 25 wt.%, or combined with NACP fillers, 12.5 wt.% each. The dentin shear bond strength in both formulations was higher than the control. The film thickness, flexural strength, and elastic modulus values were within the normal range. The amount of calcium and fluoride release and phosphate in the formulation containing NACP was very high and was sustained for up to 70 days. Both formulations could re-release the same amount of ions following three recharge cycles (Figure 7) [104]. Such cements may provide an advanced approach to control demineralization around fixed prostheses.

### 4.3. Bioactive Glass (BAG) Fillers

Bioactive glass (BAG) has been proven to have antibacterial properties against oral bacteria and the potential to remineralize oral hard tissues [49]. Depending on the ratio of calcium oxide to phosphorus pentoxide, the bioactivity of BAG can be controlled [105,106]. The antibacterial activity of BAG is ascribed to the part that releases ions (e.g., calcium and phosphate), which are poisonous to bacteria and induce the neutralization of the surrounding acidic environment [49]. Although the first BAG was produced over 40 years ago, research into its possible use in resin-based composites has recently begun. Thus, the precise mechanism of the BAG antimicrobial effect could have been illustrated better [49]. However, the high precipitation of Ca^2+^ and PO_4_^3−^ ions may induce a neutralization effect and indirectly interfere with the growth of cariogenic biofilms [107].

#### 4.3.1. BAG in Resin-Based Composite Restorations

In several studies, BAG was added to resin-based composite restorations to induce acid neutralization [17]. It has been shown that a resin-based composite containing BAG can fulfill the mechanical property demands needed for dental restorations [105,106]. In addition, BAG can reduce fluid movements in the tubules, thereby reducing dentinal hypersensitivity [105,106]. In one study, resin-based composites containing BAG with and without fluoride have been advocated to reduce the degradation of dentin [108]. After 30 days of storage in artificial saliva, the two resin-based composite systems reduced the solubility of C-terminal cross-linked telopeptide (ICTP) and C-terminal telopeptide (CTX), preventing dentin degradation and demineralization. Such observation may reveal BAG’s capabilities to inhibit the activation of matrix metalloproteinases (MMPs). The remineralization effect was seen following the 30 days of storage. High participation of calcium and phosphate minerals was observed over the teeth restored with the BAG-containing resin-based composite. The amount of remineralization was greater in the group containing both BAG and fluoride [108].

**Figure 8:**
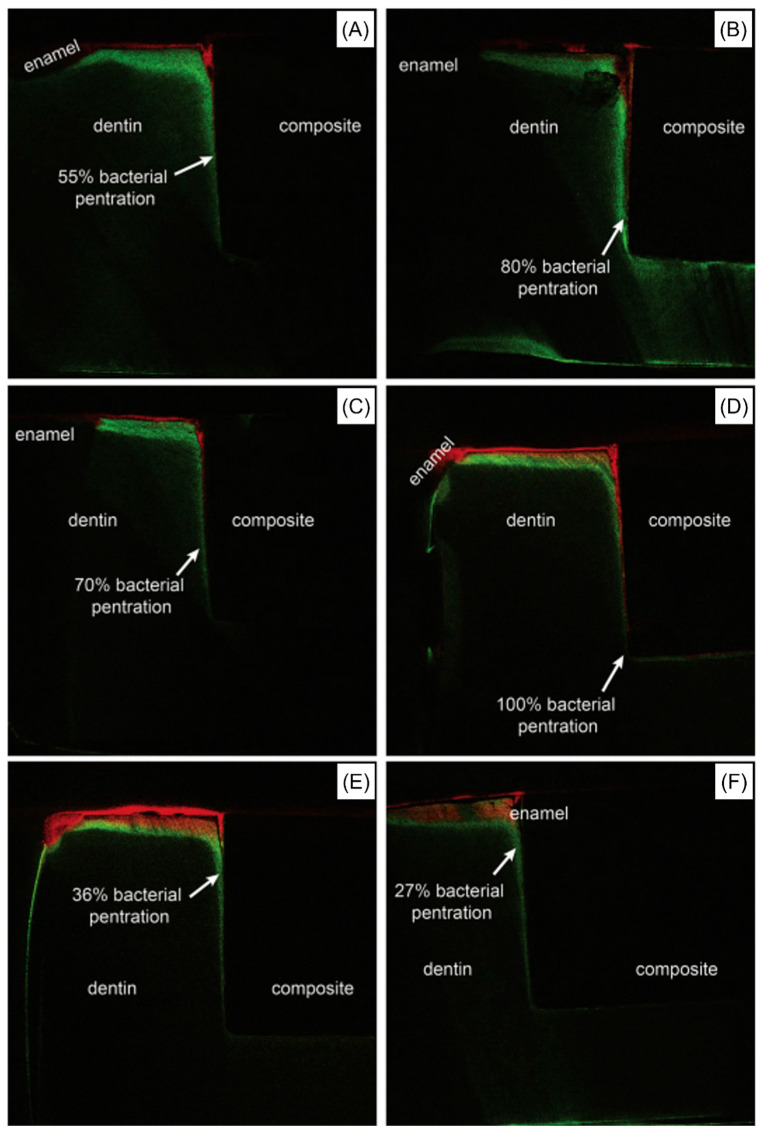
Different fluorescence images of six different samples (**A**–**F**) showing the capabilities of a resin-based composite containing 15 wt.% of bioactive glass (BAG) to prevent bacterial penetration (red color) and dentin demineralization (green color) by an average of 40%, compared to the control that allowed 100% bacterial penetration. Reprinted/adapted with permission from Ref. [49]. 2016, © Elsevier.

BAG as a filler can contribute to the inhibition of microbial activity. In one investigation, two resin-based composites, BAG-free resin-based composite as a control and resin-based composite containing 15 wt.% of BAG, were tested to see their effectiveness on gap formation and depth of the bacterial penetration relying on optical micrograph. For the control, the bacterial penetration was very deep, reaching the bottom of the cavity prepared with a high number of microorganisms [49], while the 15 wt.% BAG resin-based composites reduced the bacterial penetration by an average of 40%. (Figure 8) This implies that the release of ions from BAG can inhibit the biofilm’s growth and spread by regulating the gap environment [49].

Resin-based composite containing BAG inhibited the growth of *S. mutans* biofilms. BAG was incorporated into resin-based composites at 5, 10, and 30 wt.%., where the resin matrix was composed of BisGMA and TEGDMA at the ratio of 70:30 [109]. It was found that resin-based composites containing 10 and 30 wt.% of BAD reduced the *S. mutans* by 1- and 2-log, respectively. However, loading at 30 wt.% significantly reduced compressive and flexural strength [109]. In another investigation, a synergetic effect was observed when BAG and magnesium oxide nanoparticles were combined in a resin-based composite formulation [110]. This combination could reduce the growth of *S. mutans* biofilms. However, as in previous studies, increasing the BAG concentration negatively affected the material’s mechanical properties [110].

#### 4.3.2. BAG in Resin-Based Pit and Fissure Sealants and Dental Adhesives

Pits and fissure sealants containing BAG had high marginal adaptability and retention and a high preventive effect, which decreases bacterial infiltration in plaque stagnation areas. In one study, BAG was incorporated into resin-based sealants in different mass fractions ranging between 12.5 and 50 wt.% [111]. While increasing BAG concentrations were associated with an increased neutralization effect, the mechanical properties were significantly reduced, indicating that BAG, as most bioactive fillers, can only be incorporated in small mass fractions [111]. It was found that enamel surfaces restored with sealants containing BAG were more resistant to demineralization challenges [112].

Orthodontic adhesives might also be reinforced by BAG, which is proven to prevent the demineralization process from taking place in the oral cavity [113]. In one study, a commercial orthodontic adhesive was modified to contain 1, 3, and 5 wt.% of BAG. As the concentration of BAG was increased in the orthodontic adhesives, a greater anti-demineralization action was seen without affecting the adhesive’s biocompatibility and bonding strength. This is because the ion-buffering impact of BAGs-released ions prevents a drop in intraoral pH, which is responsible for the chemical anti-demineralization effect [113]. Another investigation illustrated that antibacterial and remineralization effects were observed when 10 to 15 wt.% of BAG was incorporated into an orthodontic adhesive [114]. It was found that around 200–300 µm of the tooth structure around BAG-containing orthodontic adhesives did not undergo demineralization, as the mineral deposition from the BAG particles preserved the tooth structure. Higher microhardness of teeth was observed when BAG-containing adhesives were used, and BAG’s incorporation did not affect the formulated adhesives’ biocompatibility and bonding strength [114].

Several reports found that orthodontic adhesives containing BAG may prevent tooth demineralization around orthodontic brackets [115,116]. In one study, BAG nanoparticles were doped with gallium and incorporated into an orthodontic adhesive of 1, 3, and 5 wt.% [117]. In a dose-dependent manner, higher Ca^2+^ and PO_4_^3−^ ion release and greater antibacterial properties were observed as the concentration of BAG-gallium increased [117]. Another investigation illustrated that 4-methacryloxyethyl trimellitic anhydride/methyl methacrylate-tri-n-butyl borane (4-META/MMA-TBB)-based resin containing various amounts (0–50%) of BAG as an orthodontic adhesive was capable of releasing high amounts of Ca^2+^ and PO_4_^3−^ ions, and preserving enamel hardness following chemical demineralization [118]. This was accomplished without losing the intended adhesive’s shear bond strength [118].

### 4.4. Hydroxyapatite (HA) and Fluorapatite (FA)

Hydroxyapatite (Ca_10_(PO_4_)_6_OH)_2_ has been utilized extensively in biomedical and dental applications because of its similarities to the primary mineral components of hard tissues in the human body, such as bone, dental enamel, and dentin, as well as its biocompatibility, bioactivity, and low solubility in moist conditions [119]. The substation of the hydroxyl group in enamel apatite could also happen with F^−^ ions, forming fluorapatite (FA) or Fluorohydroxyapatite (FHA). Fluoride is known to have superior acidic substance resistance, lower solubility, and greater hardness. Meanwhile, biocompatibility between FA and HP remained similar [120].

Early investigations incorporating HA and FA into restorative materials aimed to improve the mechanical properties of the designed materials. It was found that glass ionomer cements containing HA and FA particles were associated with higher fracture toughness and flexural and tensile strength [121,122]. When incorporated into resin-based composite formulation, HA could improve the flexural and compressive strength, with the highest strength observed at 20 wt.% loading [123]. Recently, the incorporation of HA and FA into resin-based composites was attempted as a strategy to impart bioactivity in these polymeric materials. In one study, HA nanowires were synthesized via a hydrothermal technique and soaked in dopamine [124]. Then, dopamine-coated HA was combined with silver nanoparticles and incorporated into a resin matrix composed of BisGMA and TEGDMA. Deposition of calcium and silver elements was observed over the material’s surface, which contributed to the inhibition of *S. mutans* biofilms. Increasing the dopamine-coated HA-silver concentration was associated with more biofilm reduction and greater strength [124]. Doping HA with zinc-strontium [125] or titanium dioxide [126] was also attempted as a strategy to improve the bioactivity of resin-based composite restorations.

HA was incorporated into pit and fissure sealants as a remineralization strategy [127]. Incorporating 10 or 30 wt.% of HA did not dramatically affect the bond strength, depth of cure, and degree of conversion of the formulated sealants. A high release of Ca^2+^ and PO_4_^3−^ ions was observed, and a scanning electron microscope visualized more remineralized areas over enamel surfaces adjacent to HA sealants. Orthodontic adhesives can also be treated with 5% HA nanoparticles to limit bacterial development and reduce the growth of cariogenic bacteria [128]. Another study indicates that when incorporating nano-FA or nano-FHA in a resin-based orthodontic adhesive, the fluoride release properties were increased, quadrupling the amount of fluoride after 70 days when compared to the control group [129].

### 4.5. Boron Nitride (BN)

Boron nitride (BN) has several medical applications. Due to its high chemical stability, BN has been used as an alternative to graphene and its derivatives [130]. BN also has excellent biocompatibility and functionality, giving a wide variety in pharmaceutical drug design [131]. Initial investigations utilizing BN in dentistry aimed to improve the mechanical properties of the designed material [132,133]. Its capabilities to deposit minerals have led many researchers to incorporate it for bioactivity purposes. In one study, BN nanotubes were incorporated into a dental adhesive at different mass fractions ranging between 0.05 to 0.15 wt.% [134]. The resin matrix was composed of BisGMA and HEMA in mass ratios of 66.6 and 33.3 wt.%, respectively. As BN nanotube concentration increased, greater microhardness and ultimate strength values were observed without affecting the degree of conversion. Micro-Raman spectroscopy and scanning electron microscopy images showed mineral deposition over the adhesive surfaces, indicating the capabilities of these nanotubes to remineralize the surrounding hard dental tissues [134].

**Figure 9:**
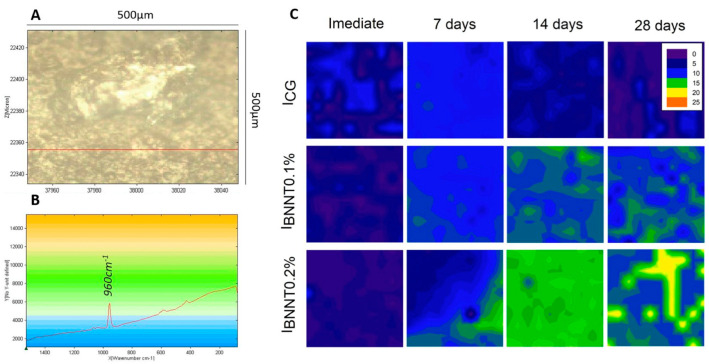
Evaluation of the mineral deposition using Raman analysis after immersion in artificial saliva for different time points. (**A**) Resin-based sealant showing the scanned surface (500 µm × 500 µm). (**B**) Phosphate ion (PO_4_^3−^) peak at 960 cm^−1^. (**C**) As in the legends, color changes from blue to orange indicate more phosphate deposition. More phosphate deposition was observed as more boron nitride nanotubes were incorporated. Reprinted/adapted with permission from Ref. [136]. 2019, Bohns et al.

BN nanosheets were modified with zinc oxide nanoparticles and incorporated into a resin-based composite formulation in another study [135]. At the load of 0.5 wt.%, BN nanosheets-zinc oxide NPs significantly reduced the *S. mutans* biofilms without affecting the material’s mechanical properties [135]. Similar results were observed when BN nanotubes were incorporated into a resin-based sealant [136]. The resin matrix comprised 90 wt.% of TEGDMA and 10 wt.% of BisGMA. Then, 0.1 and 0.2 wt.% of BN nanotubes were added and subjected to different mechanical and physical assessments. The addition of BN nanotubes did not affect the biocompatibility and polymerization kinetics of the sealants and the ultimate tensile strength. When immersed in artificial saliva, sealants containing BN nanotubes demonstrated mineral deposition in a dose-dependent manner (Figure 9) [136]. BN as a potential remineralizing agent is a new avenue in restorative dentistry. Therefore, more research papers may conduct further investigations to evaluate the different applications of this material.

## 5. Future Perspectives

Several studies discussed ion-releasing materials as a strategy to prevent the onset of secondary caries around dental restorations. While the available data is promising, further investigation will be beneficial to overcome the drawbacks of the weak material’s mechanical and physical properties and tailor different restorative materials according to the intended applications. The focus of the previous reports was to investigate the bioactivity of such formulations. This led these reports to conduct a basic mechanical evaluation of the designed formulations. It is important to realize that materials with high bioactivity would fail mechanically due to stress-induced fractures if the mechanical properties were poor [14,47,48]. Properties such as bonding strength, microhardness, compressive strength, water sorption and solubility, and color characteristics were ignored in some investigations. Structuring a comprehensive mechanical and physical evaluation in future studies will be essential to obtain more valuable information concerning the performance of such formulations [14,47,48].

It is very important to subject materials’ mechanical and physical properties for long-term evaluation. Dental restorative materials are subjected to cyclic load and fatigue inside the oral cavity due to the force of mastication and frequent exposure to oral fluids and consumable beverages [14,47,48]. As a result, restorative materials may show surface and body degradation over time, affecting the designed formulation’s integrity. The same can be applied concerning the materials’ bioactivity, as this feature might decay following aging [64]. Therefore, newly designed remineralizing formulations must undergo comprehensive evaluation at immediate testing and after actual or artificial aging.

One of the main drawbacks in the reported investigations is that the listed materials’ polymerization properties are not yet fully understood, such as the material cross-link density, degree of conversion, and depth of cure. Optimum polymerization and cross-linking are essential to ensure suitable the materials’ mechanical and physical properties [137]. Several reports illustrated that resin-based materials with under-achieved polymerization are more susceptible to clinical failure due to the high risk of resin matrix degradation that can weaken the material and facilitate biofilm adhesion [138,139,140]. Therefore, evaluating the polymerization kinetics of such materials can allow further improvement of the materials’ properties, characterization, and ion release capabilities.

Most of the reported studies tested the designed materials in vitro. Having these materials tested in a more representable environment, in situ or in vivo, is highly needed, as the complexity of the oral biofilm and the influence of host-related factors can be experimented with, and different results are expected compared to the in vitro settings [141]. Therefore, future studies may consider adopting a translational clinical setting to conduct further investigations concerning the clinical performance of ion-releasing polymeric materials inside the oral cavity.

## 6. Conclusions

A plethora of evidence suggests that implementing ion-releasing restorative materials in restorative dentistry may minimize the biological failure of these materials due to the onset of secondary caries. The most common remineralizing fillers used in resin-based material formulations were Nano-sized Amorphous Calcium Phosphate (NACP), Calcium Fluoride (CaF_2_), Bioactive Glass (BAG), Hydroxyapatite (HA), Fluorapatite (FA), and Boron Nitride (BN). Most of the reported studies focused on formulating resin-based composite formulations, with fewer reports concerning the design of resin-based sealants, dental adhesives, and crown cement. The released ions from the designed bioactive formulations may neutralize the acidity around the placed materials, restore the lost minerals from the tooth structure, and indirectly modulate the oral biofilms. In addition, synergetic antibiofilm inhibition was observed when the remineralizing compounds were combined with other bioactive compounds, such as quaternary ammonium, suggesting a dual action against the onset of secondary caries. Future investigations may consider further evaluation and characterizations of the designed materials to understand the mechanical and antibacterial performance of these materials comprehensively. Furthermore, clinical translational models are needed to test these bioactive formulations inside the oral cavity.

## Data Availability

Not applicable.
